# Fe-S Protein Synthesis in Green Algae Mitochondria

**DOI:** 10.3390/plants10020200

**Published:** 2021-01-21

**Authors:** Diego F. Gomez-Casati, Maria V. Busi, Julieta Barchiesi, Maria A. Pagani, Noelia S. Marchetti-Acosta, Agustina Terenzi

**Affiliations:** Centro de Estudios Fotosintéticos y Bioquímicos (CEFOBI-CONICET), Universidad Nacional de Rosario, 2000 Rosario, Argentina; barchiesi@cefobi-conicet.gov.ar (J.B.); pagani@cefobi-conicet.gov.ar (M.A.P.); marchetti@cefobi-conicet.gov.ar (N.S.M.-A.); terenzi@cefobi-conicet.gov.ar (A.T.)

**Keywords:** mitochondria, algae, iron-sulfur, ISC machinery, CIA machinery

## Abstract

Iron and sulfur are two essential elements for all organisms. These elements form the Fe-S clusters that are present as cofactors in numerous proteins and protein complexes related to key processes in cells, such as respiration and photosynthesis, and participate in numerous enzymatic reactions. In photosynthetic organisms, the ISC and SUF Fe-S cluster synthesis pathways are located in organelles, mitochondria, and chloroplasts, respectively. There is also a third biosynthetic machinery in the cytosol (CIA) that is dependent on the mitochondria for its function. The genes and proteins that participate in these assembly pathways have been described mainly in bacteria, yeasts, humans, and recently in higher plants. However, little is known about the proteins that participate in these processes in algae. This review work is mainly focused on releasing the information on the existence of genes and proteins of green algae (chlorophytes) that could participate in the assembly process of Fe-S groups, especially in the mitochondrial ISC and CIA pathways.

## 1. Introduction

Iron (Fe) is an essential micronutrient for all aerobic organisms. However, it is highly toxic in the free state and can cause oxidative stress and damage to cellular macromolecules. For this reason, organisms developed mechanisms to regulate the content of Fe, responding to the deficiency or increase of the metal. Fe is present in numerous proteins that participate in energy metabolism, such as those that are involved in the mitochondrial and chloroplastic electron transport chains [1].

Both Fe and S are two compounds that are important for the synthesis of Fe-S groups and ferrosulfoproteins. These groups are inorganic cofactors that are present in numerous proteins that participate in different metabolic pathways such as photosynthesis and respiration, previously mentioned, but also in the regulation of gene expression, protein translation, maintenance of DNA integrity, and in metabolic pathways related to the assimilation of nitrogen, sulfur and iron, and amino acid metabolism [2,3,4].

Although there are numerous works that characterized the function and regulation of genes and proteins that participate in the production of Fe-S groups in bacteria, yeasts, and humans, little is known about the occurrence and function of these genes in photosynthetic organisms, especially in algae. The objective of this work is to identify the distinctive characteristics of the Fe-S group synthesis pathway in chlorophytes mitochondria. For this, we have reviewed the importance, incorporation, and uses of Fe and S in these organisms and also made a survey on the presence of possible genes and proteins that would be involved in the ISC pathway of Fe-S synthesis in these algae.

## 2. Fe and S Uptake and Metabolism in Algae

Fe-S clusters are among the most ancient cofactors used by living organisms. Life originated in water, where originally its constituent elements were abundant and easily accessible. However, with the evolution of photosynthetic organisms, the atmosphere and the oceans filled with oxygen, and the bioavailability of the elements radically changed. Chlorophytes have to incorporate the substrates for the synthesis of Fe-S centers, Fe and S, from the surrounding medium. In primeval times, Fe was present as the soluble ferrous (+2) ion. However, in oxygenic environments, Fe is oxidized to ferric (+3) ion, which forms insoluble oxides and hydroxides [5]. Land plants developed two different strategies to solubilize the metal: reduction and chelation. Both are energy expensive, but it’s the price to continue taking advantage of the chemical properties of Fe for biological reactions. Most work regarding Fe metabolism in chlorophytes was done with the model algae *Chlamydomonas reinhardtii* because it is easy to grow in a synthetic defined medium, and its genome has retained genes from the last common ancestor from both the plant and animal lineages. By homology searches in genomic and transcriptomic databases, particularly from algae grown in metal deficient conditions, with known yeast, animal, and plant transporters as baits, the repertoire of Fe and other metal transporters in *Chlamydomonas* and other algae has been analyzed [6]. *C. reinhardtii* incorporate Fe by a reduction-based strategy. Insoluble ferric oxides and other salts are solubilized by the activity of ferric reductases on the plasma membrane. *Chlamydomonas* has one gene of the NOX family of ferric reductases, *FRE1*, orthologous to the *Fre1/Fre2* and *FRO2* genes involved in Fe uptake in yeast and *Arabidopsis thaliana*, respectively. FRE1 is localized to the plasma membrane and is induced by Fe deficiency, coordinately with FEA1, FEA2, FOX1, and FTR1 [7]. FOX1 and FTR1 are similar in molecular functions to the Fet3p/Ftr1p pair in yeast, and perform ferroxidation and high affinity Fe^3+^ uptake in *Chlamydomonas*, respectively [6,8]. FOX1 is a multicopper oxidase with the highest similarity to human ceruloplasmin and hephaestin, and this fact explains the copper-dependency for Fe assimilation in this alga, like in yeast [9]. The permeases like FTR1 are abundant in algal genomes and are thought to channel ferric ions to the cytoplasm, intimately associated with the multicopper oxidases; however, some chlorophytes do not express these proteins, nor they have the corresponding genes (see below). *Chlamydomonas* cells have an extracellular space between the wall and the plasma membrane that houses proteins involved in nutrient assimilation [10]. FEA are algal-specific proteins secreted to this periplasmic space. Although the exact biochemical and molecular role of these proteins remains to be determined, they are the major secreted proteins during Fe deficiency and are most probably involved in Fe assimilation. *Chlamydomonas* lacking cell wall secrete FEA proteins to the medium and are more sensitive to Fe deficiency compared to algae with cell walls that retain FEA proteins [7]. 

Recently *Ostreococcus tauri* has been proposed as a new model green alga for Fe metabolism studies [11]. This alga grows in open oceans where Fe scarcity is more prevalent than in *C. reinhardtii* growing niches. The most striking characteristic in *O. tauri* Fe uptake is that it does not seem to involve Fe reduction, because it lacks the classical components. *O. tauri*, and other green algae like *Ostreococcus lucimarinus* or *Micromonas* sp., do not have genes for the *FOX1/FTR1* pair [6], and although they encode ferric reductases, they are not induced by Fe deficiency [11]. Additionally, there is no clear connection between copper and Fe metabolism, and, instead, zinc seems to play an important role in regulating iron uptake [11]. Several other genes coding for potential Fe uptake transporters have been detected in *O. tauri*, including one gene from the ZIP family, related to the *Arabidopsis* IRT1 high affinity Fe uptake transporter, and a gene from the algal FEA family, which seems to include potential transmembrane and Fe binding motifs [11]. 

Once iron enters the algal cell, it must be trafficked to the different organelles, and when in excess, transported to sites of storage. Major sinks for Fe are chloroplasts and mitochondria. However, rather little is known about transport of Fe across these membranes. The mitochondrial solute carrier (MSC) family is a large family of intracellular transporters involved in the transport of multiple substrates. Mrs3p/Mrs4p from yeast belong to this family and are involved in Fe transport across inner mitochondrial membrane [12]. MITs from rice and *Arabidopsis* are also members of the same family and were recently related to Fe traffic into the mitochondria [13,14]. A protein similarity network of the MSC family led to the identification of a cluster of proteins that includes Mrs3p/4p, plant MITs and predicted iron transporters from algae [14]. 

Sulfur, the second element in Fe-S clusters, and an essential macronutrient present in proteins, lipids, carbohydrates, and several metabolites, has also suffered a great change in its most abundant chemical form in the environment. In ancient oceans, it was mainly present as sulfide (S^2−^), while with the advent of oxygenic photosynthesis, it was oxidized to sulfate (SO_4_^2−^). Primary producers have to invest energy in its reduction to sulfide in order to be able to incorporate it to biomolecules. The sulfate anion (SO_4_^2−^) is the preferred source of S, but *Chlamydomonas* also has the ability to acquire sulfur from organic compounds. This is achieved by the secretion of two arysulfatases (ARS) to the periplasmic space, capable of cleaving sulfate anions from esterified organic sulfate [15,16]. The high affinity sulfate transporters SULTR2 (plant type H^+^/SO_4_^2−^ cotransporter) and SLT1 and SLT2 (bacterial and animal type Na^+^/SO_4_^2−^ cotransporters) on plasma membrane mediate SO_4_^2−^ uptake [17]. Once inside the cell, SO_4_^2−^ must be reduced in order to be incorporated in biomolecules. But because SO_4_^2−^ is a relatively inert molecule, the first step prior to assimilation is the activation by the enzyme ATP sulfurylase to render adenosine phosphosulfate (APS). In the pathway leading to reduction, the S of APS is reduced to sulfite by APS reductase, and sulfite is further reduced to sulfide by sulfite reductase. Finally, sulfide is incorporated into the amino acid skeleton of O-acetylserine (OAS) by OAS thiollyase to form cysteine [18], which could be further transformed into methionine, the methyl donor S-adenosylmethionine (AdoMet), and the antioxidant glutathione [19]. The assimilation process is essentially similar to that of *Arabidopsis*, and in all photosynthetic organisms, sulfate reduction occurs in the plastids, but ATP sulfurylases are also present in the cytosol, and plant ATP sulfurylases have a different evolutionary origin than those from green algae [20]. Cysteine, the source of S for the ISC Fe-S cluster biosynthesis, can be synthesized in the cytosol, plastids, and mitochondria in nearly all plant species. Current studies suggest a scenario in an Arabidopsis leaf where chloroplasts generate sulfide via reductive sulfate assimilation, the mitochondria provide the bulk of OAS, and the cytosol produces most of the cysteine [21]. It is unknown where the main synthesis of cysteine occurs in green algae, but *C. reinhardtii*, *Coccomyxa subellipsoidea* and *Volvox carteri* genomes contain several genes coding for OAS thiollyases [22]. 

Studies in *Chlamydomonas* have shown that transcript levels and activities of transporters and enzymes participating in the S assimilation pathway, such as ARS, SULTR2, SLT1, SLT2, ATP sulfurylase, APS reductase, and OAS thiollyase, increase under S deficiency conditions, and are highly regulated by the demand for reduced sulfur and by environmental conditions [23].

## 3. Synthesis of Fe-S Clusters

The most common and simplest forms of Fe/S clusters are of the (2Fe-2S) and (4Fe-4S) type, but also (3Fe-4S) forms or more complex clusters containing additional heavy metal ions are known [24,25]. Specifically, the iron-sulfur group biogenesis pathways include several proteins involved in apoprotein maturation in different cellular compartments [26].

Aerobic eukaryotic organisms depend on mitochondria to synthesize Fe-S groups, while photosynthetic aerobic organisms also synthesize Fe-S groups in chloroplasts [4,27]. Although mitochondria can assemble their own Fe-S proteins, they play a crucial role in the biogenesis of cytosolic and nuclear ferrosulfoproteins [26,27].

It was described in plants, by studies carried out mainly in *A. thaliana*, that there are three metabolic pathways for the assembly of Fe-S groups: (i) the SUF pathway (mobilization of sulfur) in chloroplasts, (ii) the CIA pathway of assembly of Fe-S groups in cytosol, and (iii) the ISC pathway, mitochondrial iron-sulfur cluster [3,27]. The SUF and ISC machines carry out the synthesis of Fe-S groups in three basic stages. In the first stage, S is obtained from the reaction catalyzed by a cysteine desulfurase, NFS, and combined with Fe on a scaffold protein for de novo synthesis of (2Fe-2S) groups. In a second stage, the Fe-S group is released from the scaffold with the aid of chaperones and co-chaperones, and bound by a transfer protein. At this point, the (2Fe-2S) group can be inserted into apoproteins, trafficked to the late-acting machinery for (4Fe-4S) group synthesis, or, in the ISC pathway, used for export of a yet unknown sulfur-containing species to be utilized by the CIA system. The third stage is less known and comprises the conversion of (2Fe-2S) into (4Fe-4S) groups, and the insertion into apoproteins by dedicated targeting factors [27,28].

There are numerous works on the characterization of the synthesis pathway of Fe-S groups in mitochondria mediated by the ISC complex [29]. It has been reported that this complex is a heterodecamer composed of five proteins, NFS1, the scaffold protein ISCU, ISD11, an acyl-carrier protein (ACP), and frataxin [29].

Nfs1 is a pyridoxal phosphate-dependent mitochondrial cysteine desulfurase. This protein produces S from alanine [30]. It was shown that in plants, specifically in Arabidopsis, there are two isoforms, AtNFS1, located in mitochondria and AtNFS2, with chloroplastic localization [3,31,32,33]. ISD11 is a member of the LYR protein family, which lacks orthologs in prokaryotes but is highly conserved in yeast, humans, and plants [34,35,36], while IscU is a scaffold protein homologous to ISU from plants [37]. Three genes encoding ISU1-3 were found in Arabidopsis (At4g22220, At3g01020, At4g04080) [31,38,39], while ACP is an acyl group carrier protein, homologous to *Arabidopsis* AtACP1-3 [40]. Finally, frataxin is a highly conserved protein from prokaryotes to eukaryotes [41,42]. This protein has been involved in various processes such as Fe homeostasis, respiration, heme metabolism, oxidative stress, and transfer of persulfide groups [43,44,45,46,47,48,49]. 

### 3.1. Cysteine Desulfurases

As mentioned above, the first step in the synthesis of Fe-S groups in mitochondria and chloroplasts is catalyzed by a cysteine desulfurase [50]. These enzymes are found in the three kingdoms of life, representing a conserved group of proteins with several essential functions, such as the formation of Fe-S groups and the synthesis of biotin, thiamine, molybdenum cofactor, thionucleosides in tRNAs, and lipoic acid [50,51,52,53]. 

Cysteine desulfurases belong to the class V group of aminotransferases and are classified into two main classes (I and II) based on their sequence similarity [50,54]. A particularly recognized structural difference between type I and type II is the catalytic loop, which contains a nucleophilic cysteine. This catalytic loop of type I cysteine desulfurases is longer than that of type II enzymes and, therefore, it is considered to be structurally more flexible than that of type II enzymes [55,56,57]. 

Class I of cysteine desulfurases contains the consensus sequence SSGSACTS and group II, the sequence RXGHHCA. In general, the group I contains the enzymes with sequence similarity to NifS and Isc and those of class II, those enzymes related to SufS and CsdA [50]. The resolution of the crystal structures of the IscS cysteine desulfurases of *Escherichia coli* (EcIscS) and *Thermotoga maritima* (TmNifS), showed that these proteins form homodimers of around 90 kDa, also showing high structural similarity [55,58]. Each monomer is composed of a major domain in which the PLP cofactor is bound to a conserved lysine, and a minor domain that houses the active site cysteine [55,58,59]. 

The first cysteine desulfurases were described in the nitrogen-fixing bacteria *Azotobacter vinelandii*, called IscS and NifS. IscS is the cysteine desulfurase of the ISC system for the synthesis of Fe-S centers, while NifS is exclusively involved in the maturation of the enzyme nitrogenase [50,60]. In bacteria like *E. coli*, three genes were found that code for cysteine desulfurases: IscS, SufS (CsdB) and CsdA (CSD) [50]. On the other hand, in humans and in *S. cerevisiae*, there is a single gene that codes for a type I cysteine desulfurase called Nfs1 [61,62]. In humans, there are two isoforms of Nfs1 located in the cytosol and mitochondria, generated by the differential use of codons at the beginning of translation [61]. In yeast, due to differential cleavage of the signal peptide, Nfs1 is mainly localized in mitochondria but is also found in the cytosol and nucleus [63]. The deficiency of Nfs1 in both organisms produces a decrease in the activity of Fe-S proteins generating serious problems in growth [62,64]. 

Few cysteine desulfurases have been described in higher plants, such as those from *A. thaliana* and *Glycine max* [65,66]. In *Arabidopsis*, the presence of AtNFS1, located in mitochondria, and AtNFS2, present in chloroplasts was described [31,33,66,67,68]; while in *G. max* the presence of four cysteine desulfurases was reported [65]. The analysis of the amino acid sequences of AtNFS1 showed that this protein belongs to class I of cysteine desulfurases [33,68]. Homology modeling studies showed that AtNFS1 exhibits the characteristic folding of class I cysteine desulfurases such as IscS from *E. coli* [32]. AtNFS1 and AtNFS2 are essential for the plant since homozygous mutants are lethal, and the reduction in the abundance of these proteins produces severe consequences in development. Furthermore, there is an additional cysteine desulfurase called ABA3 involved in the formation of the molybdenum cofactor in the cytosol, which is not related to the biosynthesis of Fe-S and groups [69]. 

Currently, the genes and proteins that would participate in the synthesis of Fe-S groups in algae have not been identified or characterized. Thus, we performed a bioinformatic search for sequence identity for NFS1 and NFS2 in Chlorophytes using the Uniprot Database [70] and the *A. thaliana* sequences as a query. The search results showed a total of eighteen sequences for NFS1 and ten sequences for NFS2 in green algae. NFS1 homologs present a percentage identity between 62.2 and 70.6% with respect to AtNFS1, with a sequence length between 406 to 488 amino acid residues (Table 1). These sequences were analyzed in order to verify the conservation of critical amino acids for the function of the enzymes. For NFS1 homologs, the residues belonging to the active site of the enzyme are almost completely conserved (T124, H152, K153, N203, E204, K245 and Y386, according to the numbering of AtNFS1) as well as the catalytic residue Cys 377 and R403, involved in substrate binding. The active site of AtNFS2 has also almost all conserved residues belonging to the active site (T144, H172, H173, N224, V225, Q252, K275), a catalytic cysteine at position 418 (C418) and an arginine that binds the substrate (R433, all positions according AtNFS2 numbering). The exceptions are those enzymes belonging to *Chlorella variabilis* and *Chlamydomonas eustigma*, where V225 is replaced by a methionine, and in the protein from *Monoraphidium neglectum*, where we found a threonine in that position. 

The presence of multiple cysteine desulfurases was previously described in other organisms such as *Bacillus subtilis* and other gram-positive bacteria. However, this had not been described in photosynthetic organisms [71]. We found two putative NFS1 proteins in *C. reinhardtii*, *C. variabilis*, *O. tauri* and *M. commoda*, whereas one NFS1 was identified in all the other algae species (Table 1). The presence of multiple NFSs in these algae could be a strategy to distribute sulfur towards different metabolic pathways. It would also be of great interest to study the possible participation and functions of these multiple mitochondrial cysteine desulfurases in the synthesis pathways of Fe-S groups in the four algae species mentioned above. On the other hand, since one of the *B. subtilis* cysteine desulfurases was reported to have reactivity towards cysteine in the presence of a sulfide transferase (SufU), it is possible that some of these desulfurases also interact with other proteins to fulfill their biological function. 

To analyze the cell location, we use the Deep-Loc1.0 server [72]. The proteins found in *C. sorokiniana*, *M. conductrix*, *T. obliquus*, *V. carteri* f. nagariensis, *O. tauri* (*2*), *R. subcapitata*, *C. primus*, *M. commoda* (*2*), *C. reinhardtii* (*2*) and *C. variabilis* (*2*), would present mitochondrial localization while in *B. prasinos* the protein would be located in plastids. The analysis for the sequences of *A. protothecoides*, *C. subellipsoidea*, *M. commoda*, *M. neglectum*, and *M. pusilla* showed a cytoplasm localization.

### 3.2. Scaffold Proteins 

As described above, plant mitochondria use the ISC pathway for the synthesis of Fe-S groups [3,27,31]. After the action of the cysteine desulfurase, the persulfide group is transferred to scaffold proteins such as ISU that participate in the maturation of the cofactor. Alignment of ISU amino acid sequences from plants, human, bacteria and yeast had shown a high conservation of these proteins through evolution [73]. We found that *A. thaliana* ISU1 (AtISU1) amino acid sequence shares 74.9% sequence identity with EcIscU, 75.4% identity with ScISU1, and 78.9% identity with human ISCU1 [39,73]. 

In yeast, it was reported that ISU expression led to a growth restoration of the *Saccharomyces cerevisiae* Δ*isu1*Δ*nfu1* thermosensitive mutant [73]. Frazzon et al. (2007) identified the presence of three AtISU proteins in *Arabidopsis* plants showing mitochondrial localization [66]. Moreover, through the analysis of *Arabidopsis* lines in which *AtISU* genes were up or downregulated, it was proposed that the three AtISU proteins contribute to the assembly of (Fe-S) clusters within mitochondria, and this is crucial for normal plant growth and development [66]. 

Functional interactions have been described among AtISU1 and other proteins form ISC machinery, for instance, with frataxin in humans and yeast [74,75,76,77,78]. More recently, our group reported that AtISU1, together with AtHSCB chaperon interacts with the Hsp70-type chaperon AtHscA2 and modulate its ATPase activity [79]. 

As for the other homologous proteins of the Fe-S group synthesis pathway, it has only been described the presence of four ISU genes in *Chlamydomonas* [80]. Thus, in order to know the existence of possible proteins orthologous genes in other chlorophytes, we performed an amino acid sequence similarity search using the sequence of *Arabidopsis* AtISU1 as a query in the Uniprot Database [70]. We identified a total of twenty-seven sequences for chlorophytes with 82.1 to 77.8 % of identity to AtISU1, all of them not functionally characterized (Table 1). We only found one ISU sequence for most of the analyzed species, except for *D. salina*, *M. commoda* and *O. tauri*, all of them containing two copies of putative ISU proteins. 

Twenty-five of these ISU sequences analyzed had between 118 and 174 amino acid residues. All of them present the three conserved cysteines that have been reported to be essential for Fe–S cluster binding in yeast ISU1 and bacterial IscU proteins [80]. The shortest protein of all analyzed, *Helicosporidium* ISU, had 91 amino acid residues due to a shorter N-terminal region and, as a consequence, lacked one of the conserved cysteines. It has been reported that mutations at the cysteine residues of *S. cerevisiae* ISU1 resulted in a loss of function of a *sod*1^−^ deficient mutant [39,81]. The longest ISU homolog analyzed, from *M. conductrix*, had 615 amino acid residues due to an extra RCC2 like N-terminal domain, proposed to be involved with cell cycle control. 

Besides, the LPPVK motif, responsible of interaction with the HscA-like chaperones [73], is rigorously conserved in all analyzed ISU proteins suggesting a similar interaction between these proteins in algae. The bioinformatic analysis in Supplementary Materials of its secondary structure showed five putative alpha helices: (the longest of 27 residues) and four beta sheets. Moreover, most of them would have mitochondrial matrix localization, that is the same location as ISU proteins in *Arabidopsis* and yeast, with the exception of ISU proteins from *A. protothecoides*, *C. variabilis*, *D. salina* (KAF5839176), *M. commoda* (XP_002507274.1), *Helicosporidium* sp. and *Micractinium conductrix* that would have cytoplasmic localization (Table 1) [72,80].

NifU proteins present in nitrogen-fixing bacteria were reported to contain a C-terminal domain called NFU. Subsequently, NFU proteins were found in humans, yeasts, cyanobacteria, and plants and were shown to be involved in the synthesis of Fe-S clusters, specifically the (4Fe-4S) forms [38,82,83,84]. Their molecular function is not completely clear. NFU1 is a late-acting factor in the biogenesis of human mitochondrial iron-sulfur proteins. It was proposed that NFU1 is an “assembler” of (4Fe-4S), able to convert two (2Fe-2S) groups bound to transfer proteins of the ISC pathway into a (4Fe-4S) group [85]. More recently, the (4Fe-4S) bound form of ISCU (human homolog of ISU proteins) was reported to physically interact with NFU1, and to transfer the already formed cubane Fe-S group to the latter [86]. The existence of three NFU proteins in plant chloroplasts (NFU1-3), and two proteins (NFU4 and 5) in mitochondria have been described [38]. NFU proteins were reported to have a highly conserved domain CXXC, containing two cysteines involved in the binding of the Fe-S cluster and thus, essential for their biological function [87].

We performed a bioinformatic search for sequence identity to NFU in Chlorophytes using the Uniprot database [70]. For this, the *A. thaliana* NFU sequences were used as a query (Table 1). When AtNFU4 was used as a query, fifteen sequences were obtained in green algae with a percentage identity between 52.9% (*B. prasinos*) to 67.5% (*C. subellipsoidea*). Using the AtNFU5 sequence as a query, the results showed a total of fourteen sequences in green algae with a percentage identity between 53.3% (*A. protothecoides*) to 68.8% (*T. obliquus*). However, many of the sequences found were the same due to the high percentage of identity between plant proteins. Thus, it was observed that thirteen sequences, from *T. obliquus*, *V. carteri* f. nagariensis, *C. sorokiniana*, *C. eustigma*, *C. reinhardtii*, *T. socialis*, *A. protothecoides*, *M. commoda*, *B. prasinos*, *O. tauri*, *C. variabilis*, *C. subellipsoidea* and *O. lucimarinus* were the same using both, AtNFU4 and AtNFU5 as a query (Table 1). The length of these sequences varied between 206 and 627 amino acid residues.

To analyze the cell location, we use the Depp-Loc1.0 server [72]. The analysis showed that the 13 proteins would have mitochondrial localization and would be found in soluble form.

On the other hand, when AtNFU1 sequence was used as a query, the results showed a total of 33 sequences in green algae with a percentage identity between 34.3% (*M. commoda*) and 57.9% (*C. subellipsoidea*). The length of the proteins found was between 162 to 1391 amino acid residues. Moreover, the analysis of the cellular localization showed that 18 of the 33 proteins found would be located in chloroplasts and of these, 5 would be found associated with the membrane.

### 3.3. Regulatory Proteins 

Another protein described to be involved in Fe-S cluster assembly in mitochondria is ISD11. This is a protein that is part of the ISC machinery and it is essential for the formation of iron sulfur clusters. ISD11 is highly conserved in eukaryotes. However, no homologs were found in bacteria [88]. In humans, it was described that Isd11, a member of the LYR protein family, is a small chaperone that binds and stabilizes Nfs1 [88,89]. In yeast, it was shown that it is essential for viability of cells. When Isd11 was depleted, accumulation of iron in mitochondria and reduced protein levels and activities of Fe/S proteins were observed, affecting mitochondrial and also cytoplasmic proteins [88]. The loss of the enzymatic activity was due to the lack of their Fe/S clusters. Thus, it was postulated that Isd11 is required for an early step in this process as it is present in a tight complex with the cysteine desulfurase Nfs1. In the absence of Isd11, Nfs1 tends to aggregate and lose its function. From this data it was proposed that Isd11 would stabilize Nfs1 and mediate the assembly/interaction of Nfs1 with other proteins [90]. 

We recently reported in *Arabidopsis* that AtNFS1 and AtISD11 regulate the ferrochelatase activity of AtFH in vitro [91]. In addition, we suggested that AtFH, AtNFS1 and AtISD11 form a multiprotein complex in *Arabidopsis* mitochondria. We found that AtFH and AtISD11 modulate the desulfurase activity of AtNFS1, indicating that the complex would have an important role in the early stages of Fe-S cluster synthesis [36]. Moreover, this complex would act in mitigating plant mitochondrial oxidative damage. In AtNFS1 deficient plants, we found a decrease of AtISD11 and AtFH mRNA transcript levels and, when AtNFS1 was overexpressed, the transcript levels of these genes were increased too, indicating the existence of a regulatory link [92]. 

Regarding the characterization of ISD11 in Chlorophytes, there is no bibliography at present. For this reason, we decided to carry out a bioinformatic search for sequence identity in the Uniprot database [70], using the AtISD11 sequence as a query. We identified a total of six sequences for green algae with a percentage identity between 34.2 and 49.1% (*C. primus*, *O. lucimarinus*, *C. reinhardtii*, *T. socialis*, *V. carteri* f. nagariensis, and *C. subellipsoidea*), all of them not yet characterized. By refining the search and using the detected *Chlamydomonas* ISD11 sequence in a second search, we found eight additional homologous proteins (from *M. commoda*, *M. pusilla*, *O. tauri*, *R. subcapitata*, *T. obliquus*, *C. sorokiniana*, *H. lacustris*, and *M. conductrix*). Interestingly, all the species show a unique ISD11 protein, except for *C. subelipsoidea* that contains two isoforms.

The fourteen protein sequences analyzed showed a length of between 84 and 135 amino acid residues, being the *T. obliquus* protein the longest of all. All of them would have mitochondrial localization, with a transit peptide of approximately 20 residues in length and would be found in a soluble form, not anchored to membranes [72]. Subsequently, we analyzed their secondary structure with bioinformatic methods [93] and we detected three possible alpha helices: the first two of about 15–17 and the third between 25–28 residues. It was also observed that in the *T. obliquus* ISD11 sequence there would be a beta strand at position 81–84 and a fourth alpha helix between residues 93–102.

When we performed an alignment by Clustal Omega [94] of all sequences of Chlorophytes plus the homologs from *Arabidopsis* and corn, we detected that three of the critical residues for the interaction with AtNFS1 are absolutely conserved (Phe38, Leu61 and Arg66) [36]. The Arg66 residue is homologous to Arg68 in humans and it has been shown that the mutation of this amino acid, although it does not prevent the formation of the NFS1-ISD11 complex, causes a severe decrease in NFS1 activity [95].

### 3.4. Chaperones and Co-Chaperones

As mentioned before, other proteins involved in the Fe-S biosynthetic pathway within mitochondria are members of a molecular chaperone system, comprising by Hsp70-type chaperones and a DNA-J-like co-chaperone (J-protein). Both proteins are involved in the maturation of the Fe-S cluster after its insertion into apoproteins [58,96].

*E. coli* contain two proteins that participate in a specialized chaperone system involved in the Fe–S cluster formation: an Hsp70 (HscA), and a J-protein (HscB), that facilitate the Fe–S cluster delivery mechanism in an ATP-dependent manner [97]. As *E. coli*, *S. cerevisiae* contain a similar chaperone system (Ssq1/Jac1), localized in the mitochondrial matrix [97,98]. It was postulated that HscA recognizes and interacts with the conserved LPPVK peptide loop of the scaffold protein IscU, and this interaction is mediated by HscB [2]. It was reported that this chaperone system is conserved in all the eukaryotes [99]. 

In *Arabidopsis*, two genes coding for mitochondrial Hsp70-type chaperons were identified: AtHscA1 (At4g37910) and AtHscA2 (At5g09590). It was postulated that these genes arose possibly from a duplication event from a gene encoding a multifunctional Hsp70 protein [100]. Moreover, a Jac1 homolog was also described (AtHSCB, At5g06410) [3]. Xu et al. (2009) reported that AtHSCB and AtISU1 can stimulate the ATPase activity of AtHscA1, suggesting that this protein is involved in the Fe–S cluster synthesis in mitochondria [101]. As mentioned, we demonstrated that AtHscA2 interacts with AtISU1 and AtHSCB, and this interaction modulates the activity of AtHscA2 [79]. On the other hand, we recently demonstrated that AtHSCB would have an additional role to that of participation in the synthesis of Fe-S groups. Using overexpressing and knock-down *A. thaliana* mutants, we suggest that AtHSCB have a relevant role in plant Fe metabolism because it would be involved in the translocation of Fe from root to shoots [102]. 

To identify the putative HscA protein in chlorophytes, we searched for homologous in the NCBI Protein Database [103] using *Arabidopsis* AtHscA1 (At4g37910) protein sequence as a query. We identified at least twenty-one sequences for chlorophytes with 70.2–89.1% of identity (Table 1). While most of the organisms contain only one copy, we identified three copies of putative HscAs in *D. salina*. It is important to note that the analysis of the amino acid sequence alignment did not allow to differentiate between HscA1 and 2 in the algae sequences.

Regarding HSCB, a bioinformatic search was performed using the NCBI Protein Database [103] and the AtHSCB homolog from *A. thaliana* (Q8L7K4) as template, limiting the search to Chlorophytes. As a result, twenty sequences were obtained (Table 1) with only one homolog present in each organism. A second search was performed in the Phytozome Database [22] using the HSCB homolog from *C. reinhardtii* (A8IK66) as template, obtaining two more HSCB sequences from *D. salina* and *Micromonas* sp. *RCC299*. The analyzed sequences presented a percentage of identity between 25.3–43.5% and a length between 144 and 318 amino acid residues, being that from *C. reinhardtii* the longest one. Most sequences were predicted to have a mitochondrial localization and a soluble form. As exceptions, HSCBs from *C. variabilis*, *H. lacustris* and *Trebouxia* sp. *A1–2* were predicted to have a cytoplasmic localization, whereas those HSCBs from *C. eustigma* and *A. protothecoides* would have a plastidic localization. Moreover, the homolog from *M. neglectum* would have a mitochondrial localization but associated with membranes, and finally, the HSCB from *G. pectorale* would have a dual localization in nucleus and cytoplasm. This potential characteristic of association of some proteins of the ISC pathway with membranes is little known and could indicate a different degree of organization of the Fe-S group synthesis pathway in some of these organisms.

It was shown that the mitochondrial cysteine desulfurase complex from humans and yeast (LYRM4/NFS1 and Isd11/Nfs1, respectively) binds ACP (acyl carrier protein) as an important subunit. This NFS1-ISD11-ACP complex forms the core of the iron-sulfur (Fe-S) assembly complex and associates with ISCU and frataxin to synthesize Fe-S clusters [104]. In higher plants, especially in *Arabidopsis thaliana*, there are three genes encoding mitochondrial ACPs namely mtACP1 (At2g44620), mtACP2 (At1g65290), and mtACP3 (At5g47630), and five plastidial ACPs, AtACP1 (At3g05020), AtACP2 (At1g54580), AtACP3 (At1g54630), AtACP4 (At4g25050), and AtACP5 (At5g27200) [40]. 

It was reported that ACP participates in the synthesis of fatty acids and lipoic acid in many organisms. In the case of algae, as well as for plants, the characterization of ACP was also focused on its role in lipid metabolism [105]. For example, Blatti et al. (2012) have demonstrated that the protein-protein interactions between the fatty acid ACP and thioesterase govern fatty acid hydrolysis within the *C. reinhardtii* chloroplast [106]. In this work, it was reported the presence of a mitochondrial ACP but do not address its participation in the assembly of iron sulfur complexes as in plants.

Following the scheme for the search for homologous proteins that we used previously, we use the three Arabidopsis mtACP sequences as a query. Subsequently, we selected an algae sequence homologous to each mtACP and performed a second search for sequence identity to expand the number of candidates. Thus, we found fourteen sequences homologous to mtACP1, sixteen to mtACP2 and eighteen to mtACP3. However, many of them were the same proteins, due to the high identity between the three plant proteins (55.7% between mtACP1 and 2 and higher than 37% for mtACP3 with the mtACP1 and 2). Therefore, nineteen protein sequences of the following Chorophytes were selected: *C. eustigma*, *B. prasinos*, *O. tauri*, *C. reinhardtii*, *T. sociales*, *G. pectorale*, *V. carteri* f. nagariensis, *M. neglectum*, *R. subcapitata*, *T. obliquus*, *C. sorokiniana*, *C. variabilis*, *Helicosporidium* sp., *C. subellipsoidea*, *M. comoda*, *M. pusilla*, *A. protothecoides*, *M. conductrix*, and *O. lucimarinus* (Table 1). 

The identity percentages with mAtACP1 varied between 43.8% for *O. tauri* and 55.6% for *C. subellipsoidea*; between 48.4% to 60.2% (for *V. carteri* and *M. pusilla*, respectively) for mtACP2 and between 30.6% to 41.7% (for *O. tauri* and *M. neglectum*, respectively) for mtACP3. 

Seventeen of the Chlorophytes protein sequences analyzed showed a length between 88 to 144 amino acid residues, being the *M. pusilla* protein the smallest. In contrast, two sequences, one belonging to *C. sorokoniana* and the second from *M. conductrix*, showed a greater length of about 440 amino acids. Eighteen proteins showed a potential mitochondrial localization, with a transit peptide of approximately 30–40 residues in length and would be found in a soluble form, not anchored to the membrane [72], whereas *M. pusilla* ACP would have a cytoplasmic localization. However, as it is a predicted sequence and the shortest one, there could be an annotation error, and the sequence may not be complete. 

The ACP residues which interact with ISD11 are conserved in all the algae sequences analyzed, suggesting the possible formation of the same subcomplexes in Chlorophytes. In addition, we detected the absolute conservation of the serine residue, responsible for binding to 4′-phosphopantethiene (4′-PPT, prosthetic group covalently attached) in all algae sequences. Recently, Cory et al. (2017) proposed a possible role of ACP in eukaryotic Fe–S cluster biosynthesis by identifying ACP–lipid interactions that promote the stability of ISD11. This could suggest that the contribution of ACP to the Fe-S assembly complex is a moonlighting function. Due to the finding of homologous sequences and structures in chlorophytes, these functions would be evolutionarily conserved.

### 3.5. Frataxin 

Frataxin is a nuclear-encoded mitochondrial protein highly conserved in prokaryotes and eukaryotes. Its deficiency was initially described as the phenotype of Friedreich’s ataxia, an autosomal recessive disease in humans, commonly resulting from a GAA expansion in the frataxin gene [43]. In yeast, the decreased expression of the frataxin homolog, Yfh1, resulted in respiratory deficiency and mitochondrial iron accumulation [45]. Furthermore, in the absence of Yfh1p, it was observed a decrease in the activity of several Fe-S-containing enzymes such as aconitase and succinate dehydrogenase, indicating a role of frataxin in the formation of Fe-S clusters and Fe-S proteins [45].

It was described that in *Arabidopsis* plants, there is a single frataxin homolog (AtFH) with dual localization in mitochondria and chloroplasts; while, in maize plants, we described the presence of two isoforms, ZmFH1 and ZmFH2, each protein also located in both plant organelles [41,44,107,108,109,110,111]. In plants, we also showed that frataxin is essential for the activity of Fe-S proteins such as aconitase and succinate dehydrogenase [44]. Furthermore, AtFH has ferrochelatase activity and is involved in the metabolism of the heme group and the synthesis of hemeproteins in mitochondria [91,112]. Moreover, we recently demonstrated that AtFH interacts with AtISD11 and AtNFS1, forming a complex that would modulate ferrochelatase activity and cysteine desulfurization by AtNFS1 [36,91].

In order to have a better understanding of frataxin function in algae we performed a bioinformatic search in NCBI using Uniprot database [70] and the amino acid sequence of the frataxin homolog from *A. thaliana* (NP_192233.2) as a template. As a result of this search ten frataxin homologs with a percentege of identity between 33% to 63% were identified. Only one homolog sequence from each organism was obtained, with the exception of *O. tauri*, in which two isoforms of frataxin were found (Table 1). By refining the search using the detected frataxin sequence from *C. reinhardtii* (A0A2K3D5B5) as a template, ten more sequences were obtained in the Phytozome Database [22] with a percentage of identity between 49.2% and 80.1% (*V. carteri* f. nagariensis, *G. pectoral*, *R. subcapitata*, *T. socialis*, *C. sorokiana*, *C. eustigma*, *C. subellipsoidea*, *C. primus*, *A. prototheicoides*, *M. commoda*) all of them not characterized. 

The twenty protein sequences analyzed presented a length between 110 and 290 amino acid residues, being that from *C. variabilis* the longest one. Two types of localization were found: cytoplasmic (for *V. carteri*, *G. pectorale*, *C. eustigma*, *C. subellipsoidea*, *M. pusilla* and *O. tauri* isoform 2) and mitochondrial (*C. reinhardtii*, *R. subcapitata*, *T. socialis*, *C. primus*, *M. commoda*, *D. salina*, *O. lucimarinus*, *O. tauri* isoform 1, and *E. siliculosus*). As an exception, the sequence of *C. variabilis* presented a nuclear localization. All sequences were predicted to be found in a soluble form [72]. Furthermore, we analyzed their secondary structures with NetSurfP-2.0 [93] and we detected the presence of 2 alpha helices and 6 beta strands, suggesting a structural conservation of these proteins with the human and yeast frataxin, whose 3D structures were determined [113,114]. Moreover, a detailed Conserved Domain (CD) search analysis of the identified sequences was performed with NCBI [115]. Results showed that all the protein sequences contain a frataxin-like domain and a putative iron binding site with significantly high conservation of aminoacidic residues, which confirms the high structural similarity that frataxin homologs contain with each other.

### 3.6. CIA Pathway

As described above, the ISC machinery drives the formation of Fe-S clusters in mitochondria and is essential for the maturation of Fe-S proteins in this organelle. In contrast, biogenesis of cytosolic Fe-S proteins requires components that are exported from the mitochondria that are later matured in the CIA system (cytosolic iron–sulfur protein assembly machinery) [116,117]. This system is conserved in virtually all eukaryotes and is essential for cell viability. Most of the CIA components were first identified and characterized in yeast and human cells and more recently, in *Arabidopsis thaliana.* Bernard et al. [118] published in 2013 an interesting work in which they summarize the identified loci, the mutants studied and the phenotypes observed for each component of the CIA pathway from *A. thaliana* plants. However, as with the ISC pathway, little is known about the occurrence, conservation, and possible proteins involved in the CIA pathway in algae.

Thus, using the same scheme that we used to identify homologous proteins of the ISC pathway, we carried out a search for homologous proteins that would participate in the CIA pathway. When conducting the search based on the CIA proteins described in *A. thaliana*, we found that, apparently, this pathway would be less conserved than the ISC one, since we could not identify the presence of many CIA homologs in algae. Table 2 shows the homologs found in chlorophytes for NDOR1 and DRE2, which are involved in an electron transfer in early steps of cytosolic Fe-S cluster biogenesis; NBP35, a scaffold protein involved in the formation of (4Fe-4S) groups; NAR1, which may play a role in the transfer of Fe-S clusters into apoproteins; CIA1 and MMS19, involved in the transfer and insertion of Fe-S clusters into target proteins; and AE7, identified as a central member of the CIA pathway [117,118].

We were only able to identify all the components of the CIA pathway in *C. reinhardtii*, but with some peculiarities. For the NAR1 protein, we found three different homologs in this alga. With respect to AE7, while it has been described three proteins in *Arabidopsis* (AE7 and two AE7-like proteins), we found only one homolog in *C. reinhardtii* [118]. Finally, except for DRE2 of *T. obliquus*, where we found the presence of two homologs, we have not identified the presence of multiple homologs of the CIA pathway in the algae analyzed (Table 2).

## 4. Conclusions

The search in genomic and protein databases showed a high conservation of the genes and proteins involved in the synthesis of Fe-S groups in algal mitochondria. Curiously, despite being unicellular organisms, and that a priori it would be thought that the pathway for the synthesis of Fe-S groups could be of less complexity compared to higher plants, many algae showed the presence of multiple genes and/or homologous proteins that would be members of the ISC pathway. In contrast, in most of the algae analyzed, we observed the presence of a single isoform of the proteins of the CIA pathway, in addition to the absence of many homologs in most of the organisms evaluated, with the exception of *C. reinhardtii*. 

Previously, the presence of some genes that would be in multiple copies in plants has been described, such as ISU1-3, ACP1-3 in *Arabidopsis* mitochondria. However, the genes encoding AtNFS1, AtISD11, AtHSCB, and AtFH were found in a single copy, although in the case of AtFH, we demonstrate dual localization in mitochondria and chloroplasts. As mentioned above, we also recently identified and characterized the presence of two genes that code for frataxin in corn, ZmFH1, and ZmFH2, both with dual localization in mitochondria and chloroplasts. While much is known about the role of frataxin homologs in mitochondria, their possible roles in chloroplasts and possible interactions with proteins in that compartment are unknown. The potential dual localization and/or the association with membranes, as well as the functions of many of the genes of the ISC pathway in algae is a subject that deserves to be studied. In addition, the presence of multiple genes found in the genomes of many chlorophytes indicates that the ISC pathway would have particular and specific characteristics in each species. This could be related to the needs of both iron and sulfur for each organism, and also with the ability to adapt to growth in different environments.

## Figures and Tables

**Table 1:** Orthologs proteins involved in Fe-S cluster synthesis in mitochondria of Chlorophytes.

**ISC Proteins**	**NFS**	**ISU**	**NFU**	**ISD11**	**HSCA**	**HSCB**	**ACP**	**FRATAXIN**
**Function**	Cysteine desulfurase	Scaffold protein	Scaffold protein	Regulatory protein	Chaperone	Co-chaperone	Regulatory protein	Iron donor
***Arabidopsis thaliana*** **genes**	At5g65720.1	At4g22220	At3g20970 (NFU4) At1g51390 (NFU5)	At5g61220	At4g37910 (HSCA1) At5g09590 (HSCA2)	At5g06410	At2g44620 (mtACP1) At1g65290 (mtACP2) At5g47630 (mtACP3)	At4g03240
**UniProt**	O49543-1	O49627-1	Q9LIG6-1 Q9C8J2	Q8L9E3-1	Q8GUM2 Q9LDZ0	Q8L7K4	P53665 O80800 Q9FGJ4	Q9ZR07
**Identified Protein Sequences**								
**Organism**								
***Auxenochlorella protothecoides***	A0A087SBH3 (c)	RMZ55380.1 (c)	A0A087SIP0		A0A087SND2	XP_011400182	A0A087SCC7	RMZ53064.1 (c)
***Bathycoccus prasinos***	K8FF08 (p)	XP_007511837.1	K8F7J7		K8F1V7	XP_007510865	K8FET8	XP_007511602.1 (p)
***Chlamydomonas eustigma***		GAX73632.1	A0A250XNZ5		A0A250XIU3	GAX75929	A0A250WTW6	GAX72625.1 (c)
***Chlamydomonas reinhardtii***	PNW80545.1 PNW87417.1	XP_001693712.1	A0A2K3D318 A0A2K3D340	A8IZ28	A8IZU0 (c)	A8IK66	Q6UKY4	A0A2K3D5B5
***Chlorella sorokiniana***	A0A2P6U3X4	PRW56423.1	A0A2P6TLS6	A0A2P6U0I2	A0A2P6TM18	A0A2P6TMK0 (c)	A0A2P6TBX9	
***Chlorella variabilis***	XP_005843562.1 (c) XP_005848297.1 (c)	XP_005850086.1 (c)	E1ZCG7 (c)		E1ZMD2	XP_005845092 (c)	E1Z6T8	
***Chloropicon primus***	A0A5B8MHP9	QDZ20066.1		A0A5B8MCM2	A0A5B8MHC3	QDZ23877		QDZ21836.1
***Coccomyxa subellipsoidea***	I0Z0B3 (c)	XP_005649671.1	I0YUZ0 (c)	I0Z768	I0YWN6		I0YSD5	XP_005644009.1 (c)
***Dunaniella salina***		KAF5838253 KAF5839176 (c)			KAF5837283.1 KAF5830450.1 KAF5832491.1	KAF5832703.1		KAF5833575.1
***Gonium pectorale***		KXZ49215.1			A0A150GMI8	KXZ55931 (c)	A0A150GAM8	KXZ42691.1 (c)
***Haematococcus lacustris***		GFH16011.1		A0A699ZQI7		GFH07090 (c)		GFH14279.1 (c)
***Helicosporidium*** **sp.**		KDD73008.1 (c)			A0A059LST8		A0A059LDR7	
***Micractinium conductrix***	A0A2P6V8I3	PSC71357.1 (c)	A0A2P6V9W1	A0A2P6VEW0	A0A2P6VHZ4	PSC73872	A0A2P6VF08	
***Micromonas commoda***	XP_002506217.1 (c) XP_002501646.1 (c)	XP_002502856.1 XP_002507274.1 (c)	C1EHF7	C1E8J2	C1EGS6	XP_002505982.1	C1E2X1	XP_002504509.1
***Micromonas pusilla***		XP_003055300.1		C1MZU9	C1MP69	XP_003057174	C1N9A6	XP_003059868.1 (c)
***Monoraphidium neglectum***	A0A0D2M031 (c)	XP_013897551.1				XP_013898922	A0A0D2MT62	
***Ostreococcus lucimarinus***		XP_001415848.1	A4RUX0	A4RYZ0	A4RWG3	XP_001419576	A4S9X3	XP_001416215.1
***Ostreococcus tauri***	XP_003078141.1 XP_003081355.2	XP_022838219.1 0US42676.1	Q01C69	A0A090M6V6	Q01AH9	XP_022839639	Q00SL4	XP_022840717.1 OUS42049.1 (c)
***Pycnococcus provasolii***		GHP08054.1						GHP08944.1 (p)
***Raphidocelis subcapitata***	A0A2V0PAY6	GBF95838.1		A0A2V0PLS6	A0A2V0PIR9	GBF88744	A0A2V0NV36	GBF91763.1
***Scenedesmus*** **sp.** **NREL46B-D3**		KAF6244147.1				KAF6263471		KAF6260390.1 (c)
***Scenedesmus*** **sp.** **PABB004**		KAF8055824.1				KAF8065471		
***Tetrabaena socialis***		PNH03155.1	A0A2J8AFK7	A0A2J8A4Z1			A0A2J8A839	PNH09606.1
***Tetradesmus obliquus***	A0A383VZF3		A0A383V5U1	A0A383WNF2	A0A383W6Z2	A0A383WKP1	A0A383VDA2	
***Trebouxia*** **sp. A1–2**		KAA6417420				KAA6420365 (c)		KAA6425040.1 (p,m)
***Volvox carteri*** **f. nagariensis**	XP_002951960.1		D8TNE6	D8TVN4	D8TMR1 (c)	XP_002948275	D8U1A8	XP_002950872.1 (c)

Ref: (c) indicates cytosolic localization, (p) indicates chloroplastic localization, (p,m) indicates dual localization in plastids and mitochondria. All other proteins where no localization is indicated showed a predicted mitochondrial localization.

**Table 2:** Orthologs of the cytosol (CIA) pathway in Chlorophytes.

	**NDOR1**	**DRE2**	**NBP35**	**NAR1**	**CIA1**	**AE7**	**MMS19**
***Arabidopsis thaliana genes***	At3g02280	At5g18400	At5g50960	At4g16440	At2g26060	At1g68310	At5g48120
***UniProt***	Q6NPS8	Q8L7Z3	Q8H1Q2-1	Q94CL6	080990-1	Q9C9G6	Q0WVF8
***Function***	Part of an electron transfer chain functioning in an early step of cytosolic Fe-S biogenesis.		Scaffold protein for formation of a [4Fe-4S] cluster	May play a role in the transfer of pre-assembled Fe/S clusters to target apoproteins	Transfer and insertion of Fe-S clusters into target proteins (CIA targeting complex)	Central member of the cytosolic iron-sulfur (Fe-S) protein assembly (CIA) pathway	Transfer and insertion of Fe-S clusters into specifics target proteins
	Transfers electrons from NADPH to the Fe-S cluster of the DRE2 homolog	Facilitating the de novo assembly of a [4Fe-4S] cluster on the cytosolic Fe-S scaffold complex					
**Identified protein sequences**							
***Organism***							
***Auxenochlorella protothecoides***	A0A087SCD2		A0A087SDS5		A0A087SU81	A0A087SMH1	
***Bathycoccus prasinos***						K8EI89	
***Chlamydomonas eustigma***		A0A250XRC9			A0A250X446	A0A250X674	
***Chlamydomonas reinhardtii***	Cre12.g551850.t1.2	Cre11.g475350.t1.3	Cre12.g494400.t1.2	Cre03.g200550.t1.2 Cre03.g199800.t1.1 Cre09.g396600.t1.1	Cre08.g374400.t1.3	A0A2K3D8M0	g14594.t1
***Chlorella sorokiniana***		A0A2P6TP36	A0A2P6TVH6		A0A2P6TVY6	A0A2P6U4B7	PRW58456.1
***Chlorella variabilis***		E1ZBV5	E1ZFC7		E1Z4N9	E1ZKW8	XP_005851493.1
***Chloropicon primus***			A0A5B8MC28			A0A5B8MEG6	A0A5B8MQB3
***Coccomyxa subellipsoidea***		I0YJK0	I0Z253	I0YLU4	I0YK24	I0Z0I7	
***Gonium pectorale***		A0A150GF73	A0A150GJ11	A0A150G6L9		A0A150G9L7	
***Helicosporidium* sp. *ATCC 50920***	A0A059LKT0						
***Micractinium conductrix***		A0A2P6VI63	A0A2P6VKI3		A0A2P6V477	A0A2P6VJ54	
***Micromonas commoda***							XP_002505452.1
***Micromonas pusilla***	C1N4X9						XP_003059724.1
***Monoraphidium neglectum***		A0A0D2MN30				A0A0D2JM25	
***Ostreococcus lucimarinus CCE9901***	A4RR54		A4RRN3			A4RRB8	A4SBG2
***Ostreococcus tauri***	A0A096P7S5		A0A090M2P3			Q01GB5	XP_003083112.2
***Raphidocelis subcapitata***		A0A2V0PD30	A0A2V0P7V0		A0A2V0PR28	A0A2V0NRG7	
***Tetrabaena socialis***				A0A2J8A236			
***Tetradesmus obliquus***		A0A383VZJ9 A0A383W1G2	A0A383W531		A0A383VND8	A0A383VR66	
***Volvox cateri* f. nagariensis**	D8UCR2	D8U8M9	D8UE05	D8UBL6	D8U2P0	D8UEN0	

## Supplementary Materials

The following are available online at https://www.mdpi.com/2223-7747/10/2/200/s1, Bioinformatic Analysis: The sequence similarity searches were carried out using BLASTP against the NCBI nonredundant database (www.ncbi.nlm.nih.gov), UniProtKB database (www.uniprot.org) and Phytozome database (https://phytozome.jgi.doe.gov/pz/portal.html) using *A. thaliana* and *Chlamydomonas* Fe-S proteins sequences as query, as described in each case. The selected sequences were aligned with Clustal Omega (www.ebi.ac.uk/Tools/msa/clustalo/). Secondary structure prediction was achieved with the servers NetSurfP-2.0 (www.cbs.dtu.dk/services/NetSurfP/) and CD-Search (www.ncbi.nlm.nih.gov/Structure/cdd/wrpsb.cgi). Intracellular location was predicted using the Depp-Loc1.0 server (www.cbs.dtu.dk/services/DeepLoc/).
