# Supplementary material for: Fe-S Protein Synthesis in Green Algae Mitochondria
Source: Plants (Basel). 2021 Jan 21;10(2):200. doi: 10.3390/plants10020200 (PMC7911964; doi:10.3390/plants10020200)
Supplement: Supplementary file 1 [file plants-10-00200-s001.pdf]

Supplementary data

Bioinformatic Analysis

The sequence similarity searches were carried out using BLASTP against the NCBI nonredundant database (www.ncbi.nlm.nih.gov), UniProtKB database (www.uniprot.org) and Phytozome database (https://phytozome.jgi.doe.gov/pz/portal.html) using *A. thaliana* and *Chlamydomonas* Fe-S proteins sequences as query, as described in each case.

The selected sequences were aligned with Clustal Omega (www.ebi.ac.uk/Tools/msa/clustalo/). Secondary structure prediction was achieved with the servers NetSurfP-2.0 (www.cbs.dtu.dk/services/NetSurfP/) and CD-Search (www.ncbi.nlm.nih.gov/Structure/cdd/wrpsb.cgi). Intracellular location was predicted using the Depp-Loc1.0 server (www.cbs.dtu.dk/services/DeepLoc/).
